# miRNA 206 and miRNA 574-5p are highly expression in coronary artery disease

**DOI:** 10.1042/BSR20150206

**Published:** 2016-02-10

**Authors:** Jianqing Zhou, Guofeng Shao, Xiaoliang Chen, Xi Yang, Xiaoyan Huang, Ping Peng, Yanna Ba, Lin Zhang, Tashina Jehangir, Shizhong Bu, Ningsheng Liu, Jiangfang Lian

**Affiliations:** * Ningbo Medical Center, Lihuili Hospital, Ningbo University, Ningbo, Zhejiang 315041, P.R. China; † Zhejiang Provincial Key Laboratory of Pathophysiology, School of Medicine, Ningbo University, Ningbo, Zhejiang 315211, P.R. China; ‡ Diabetes Research Center, School of Medicine, Ningbo University, Ningbo, Zhejiang 315211, P.R. China; § Department of Pathology, Key Laboratory of Antibody Technique of Ministry of Health, Nanjing Medical University, Nanjing 210029, P.R. China

**Keywords:** biomarker, coronary artery disease, miRNA, plasma

## Abstract

Coronary artery disease (CAD) is the leading cause of human morbidity and mortality worldwide. Innovative diagnostic biomarkers are a pressing need for this disease. miRNAs profiling is an innovative method of identifying biomarkers for many diseases and could be proven as a powerful tool in the diagnosis and treatment of CAD. We performed miRNA microarray analysis from the plasma of three CAD patients and three healthy controls. Subsequently, we performed quantitative real-time PCR (qRT-PCR) analysis of miRNA expression in plasma of another 67 CAD patients and 67 healthy controls. We identified two miRNAs (*miR-206* and *miR-574-5p*) that were significantly up-regulated in CAD patients as compared with healthy controls (*P*<0.05). The receiver operating characteristic (ROC) curves indicated these two miRNAs had great potential to provide sensitive and specific diagnostic value for CAD.

## Abstract (precis)

Our studies demonstrate two miRNAs (*miR-206* and *miR-574-5p*) that are significantly up-regulated in coronary artery disease (CAD) patients as compared with healthy controls, and the two miRNAs can be potential non-invasive biomarkers for early detection of CAD.

## INTRODUCTION

Coronary artery disease (CAD) is a major public health problem worldwide, which represents the leading cause of death globally, more than any other disease [1]. CAD is caused by atherosclerosis, which is an inflammatory disease that involves multiple cell types, including circulating cells and cells in the vessel wall [2]. Despite advances in risk factor management on an epidemiological level, many individuals continue to succumb to CAD.

Various blood markers associated with increased risk for death and cardiovascular endpoints have been identified, however little have been shown to have a diagnostic impact or important clinical implications that would affect patient management so far [3]. Therefore, there is a great interest of innovative biomarkers that can assess risks for CAD and the atherosclerotic progression, as wells as therapeutic efficacy.

miRNAs, a recently recognized class of short (19–25 nt), single-stranded, noncoding RNAs, represent a group of regulatory elements, which enable cells to fine-tune complex gene expression cascades in a wide range of biological processes, such as proliferation, differentiation, apoptosis and stress-response [4–6]. miRNAs have been found in tissues, whole blood, serum, plasma and other body fluids in a stable form that is protected from endogenous RNase activity [7,8]. miRNAs can function as managers in gene regulatory networks, and they are distinct from other biomarkers because they have a pathogenic role in the disease process and are not merely byproducts of the disease state. Many of which are expressed in a tissue- and cell-specific manner [9–11]. Thus, miRNA expression signatures in tissues and blood have a potential role in the diagnosis, prognosis and assessment of therapy. In the cardiovascular system, miRNAs are not only important for heart and vascular development but also play an essential role in cardiac pathophysiology, such as CAD, myocardial infarction and heart failure [12–15], and in cardiovascular (CV) disease in general [16,17]. The purpose of the present study is to examine distinctive miRNA profiles in plasma of patients with angiographically significant CAD to that of healthy aged-matched controls.

## MATERIALS AND METHODS

### Study population

Study participants were recruited between September 2012 and December 2013 from Ningbo Medicine Center, Lihuili Hospital, Zhejiang, China. Coronary angiograms were evaluated independently by two operators, who made visual estimation of luminal narrowing in multiple segments based on the AHA/ACC classification of the coronary tree [18]. Using these data, significant CAD was defined as at least one major epicardial vessel with >50% stenosis, assessed by quantitative coronary angiography. Patients with neither detectable coronary stenosis nor atherosclerotic vascular disease were considered as healthy controls. All individuals had no cardiomyopathy or congenital heart, severe liver or renal diseases and no malignant or primary wasting disorder. The study protocol was approved by the Ethics Committee of Lihuili Hospital in Ningbo and informed written consent was obtained from all subjects.

### Samples collection and plasma isolation

Whole blood samples were collected in tubes containing anticoagulant (EDTA). Subsequently, separation of the plasma was carried out within 2 h by centrifugation at 1900 ***g*** for 10 min, followed by a 10-min high-speed centrifugation at 16000 ***g*** of blood samples. Then, the supernatant sera were stored at −80°C.

### RNA isolation

Total RNA was isolated using mirVana™ PARIS miRNA isolation kit (Ambion, Inc.) according to manufacturer's instructions, which efficiently recovered all RNA species, including miRNAs. The quantity and quality of the total RNA extracted was determined using K5600 micro-spectrophotometer (Beijing Kaiao Technology Development Co., Ltd.) and RNA integrity was determined by gel electrophoresis.

### miRNA microarray analysis

**Figure 1:**
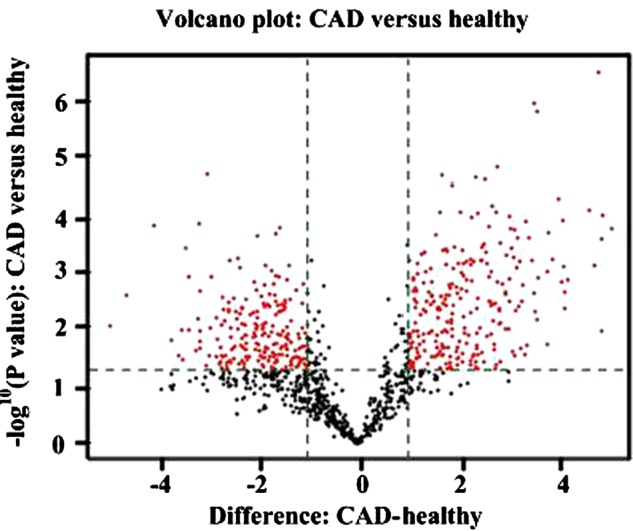
Volcano plot of all pairwise comparisons Comparisons of all miRNAs assessed in microarray analysis of RNA isolated from plasma of patients with CAD (*n*=3) or healthy volunteers (*n*=3). The volcano plot displays the relationship between fold-change and significance between the two groups using a scatter plot view. The *y*-axis is the negative log10 of *P* values (a higher value indicates greater significance) and the *x*-axis is the difference in expression between two experimental groups as measured in glog2 space. Probes identified as significant are labelled on the polt (FDR *t* test < 0.05). The red points in the plot represent the differentially expressed miRNAs with statistical significance.

**Figure 2:**
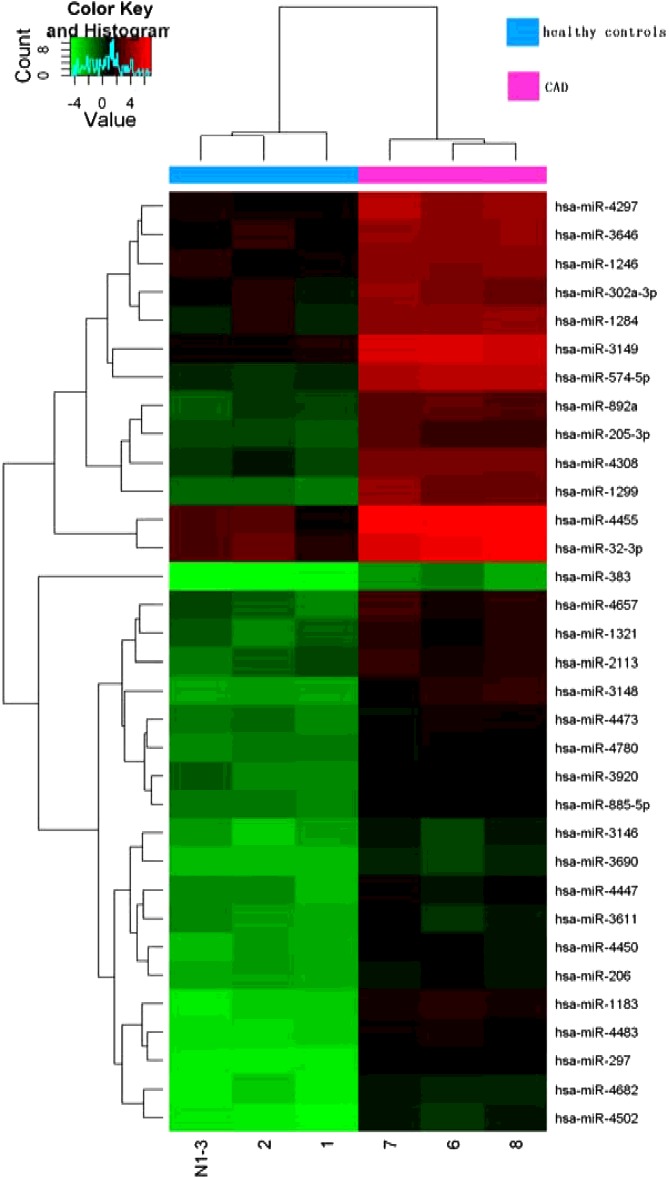
Profile of plasma miRNAs in CAD patients (*n*=3) and controls (*n*=3) Heatmap illustrates the levels of significantly changed miRNAs (fold-change > 5) in CAD patients compared with controls. Colour intensity is scaled within each row, such that the highest expression value corresponds to bright red and the lowest to bright green.

The RNA samples were labelled using the miRCURY™ Hy3™/Hy5™ Power labelling kit (Exiqon) and hybridized on the miRCURY™ LNA Array (v.18.0) (Exiqon). After washing, the slides were scanned using the Axon GenePix 4000B microarray scanner (Axon Instruments). Scanned images were then imported into GenePix Pro 6.0 software (Axon) for grid alignment and data extraction. Replicated miRNAs were averaged and miRNAs that intensities ≥30 in all samples were chosen for calculating normalization factor. Expressed data were normalized using the Median normalization. After normalization, significant differentially expressed miRNAs were identified through Volcano plot filtering (Figure 1). The threshold we used to screen up-regulated miRNAs is fold-change ≥5.0 and *P*-value ≤0.05. Finally, hierarchical clustering was performed to show distinguishable miRNA expression profiling among samples using multiple experiment viewer (MEV) software (v4.6, TIGR) (Figure 2).

### Reverse transcription reaction and quantitative real-time PCR

Extracted total RNA from isolated plasma were initially reverse transcribed using miScript® II RT Kit (Qiagen) according to the manufacturer's protocol. The RT reaction was performed at 37°C for 1 h followed by 5 min at 95°C with an iCycler system (Bio-Rad). cDNA was amplified with specific primer sets: *miR-206* (Hs_miR-206_1 miScript Primer Assay, MS00003787), (Hs_miR-574-5p_2 miScript Primer Assay, MS00043617), (Hs_miR-302a-3p_2 miScript Primer Assay, MS00009331), (Hs_miR-383_1 miScript Primer Assay, MS00004130) and RNU6 (Hs_RNU6-2_1 miScript Primer Assay, MS00033740). The amplification steps consisted of initial activation at 95°C for 15 min, followed by 40 cycles of denaturation at 94°C for 15 s, annealing at 55°C for 30 s and then extension 70°C for 34 s. Quantitative real-time PCR (qRT-PCR) was carried out on the 7500 real-time PCR system (Applied Biosystems) using miScript® SYBR® Green PCR Kit (Qiagen) according to the manufacturer's instructions.

Data were normalized for RNU6 (housekeeping gene) expression by the comparative threshold cycle method. Triplicate *C*_t_ values were averaged, the relative expression levels of miRNAs were calculated using the 2**^−^**^▵▵Ct^ method and fold-changes were calculated for each miRNA [19,20]. To evaluate PCR amplification of contaminating genomic DNA, a control without reverse transcription was included. To improve the accuracy of RT-PCR for quantification, amplifications were performed in triplicates for each RNA sample.

### Statistical analysis

Continuous data were expressed as mean ± S.D. and Student's *t* test was employed to analyse differences between two study groups. *χ*^2^ analysis was used to compare the categorical variables. The prevalence of essential hypertension, diabetes mellitus and smoking history between CAD cases and healthy controls were compared using *χ*^2^ test. A two-sided *P*<0.05 was considered to be statistically significant. We also constructed the receiver operating characteristic (ROC) curve and calculated the area under the ROC curve (AUC) to evaluate the specificity and sensitivity of CAD prediction. All the statistical analyses were performed using SPSS 13.0 software (SPSS).

## RESULTS

### Basic characteristics of the study population

In the present study, we included 67 CAD patients and 67 healthy controls. As shown in Table 1, there were no significant differences between the two groups for a series of biochemical parameters, including hypertension, diabetes, smoking history**,** age, gender, high-density lipoprotein cholesterol (HDL-C), triacylglycerols, low-density lipoprotein cholesterol (LDL-C) and total cholesterol.

**Table 1:** Clinical characteristics of the study population for plasma miRNAs profiling

	CAD (*n*=67)	Controls (*n*=67)	*P*
Male, *n* (%)	43 (64.2%)	32 (47.8%)	0.056
Smoking, *n* (%)	31 (46.3%)	25 (37.3%)	0.293
Hypertension, *n* (%)	48 (71.6%)	39 (58.2%)	0.103
Diabetes, *n* (%)	14 (20.9%)	8 (11.9%)	0.067
Mean age, years	64.70±6.79	63.69±5.96	0.359
LDL-C (mmol/l)	2.79±0.73	2.78±0.89	0.945
Total cholesterol (mmol/l)	4.22±0.95	4.36±1.23	0.409
HDL-C (mmol/l)	1.08±0.31	1.15±0.29	0.119
Triacylglycerols (mmol/l)	1.66±1.26	1.42±0.67	0.104

### miRNA profiles of plasma of CAD patients

To study the differential expression of miRNAs in CAD patients, we performed miRNA expression profiling on plasma samples from three CAD patients and three healthy controls respectively using miRCURY™ LNA Array (v.18.0) (Exiqon). In total we identified 33 miRNAs, which were differentially overexpressed in plasma from CAD patients as compared with controls. Supervised hierarchical clustering analysis of these 33 miRNAs showed distinct patterns of miRNA expression levels between CAD patients and healthy controls (Figure 2).

From these 33 miRNA, miRNAs were selected for further analysis based on following criteria: (1) at least 5-fold up-regulated in patients as compared with controls, (2) a *P*<0.01 was considered statistically significant in patients as compared with controls and (3) the bioinformatic analysis revealed the potential target genes for the identified miRNAs and these target genes might be involved in the onset and development of CAD [21–23]. Two miRNAs were showed a significant differential expression between CAD patients and controls. Compared with those in healthy controls, these two miRNAs (*miR-206* and *miR-574-5p*) in the CAD samples were increased to 8.74- and 29.53-folds respectively.

### Evaluation of miRNA expression by qRT-PCR

**Figure 3:**
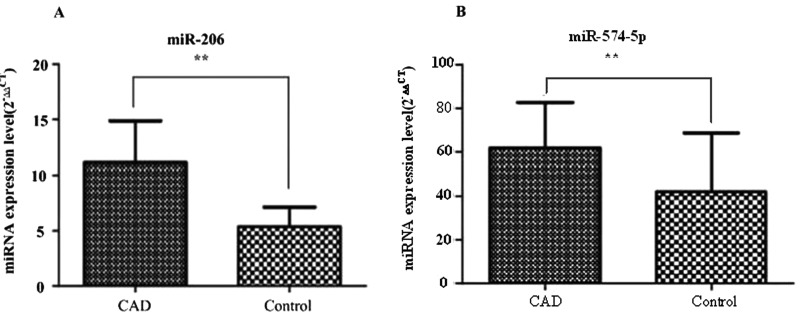
Differential expression levels of miRNAs Comparison of miRNA expression in the plasma of CAD patients (*n*=67) and controls (*n*=67) (**A** and **B**). Expression levels of selected miRNAs were analysed by qRT-PCR. Data were presented as mean ± S.E.M., **P<0.01 compared with controls.

To confirm the results from microarray-based miRNA measurements, the expression of these two miRNAs were further validated by qRT-PCR in larger cohorts comprise 67 CAD patients and 67 matched healthy controls. The expression of these two miRNAs (*miR-206* and *miR-574-5p*) remained significantly up-regulated in the CAD patients compared with the healthy controls (Figures 3A and 3B).

### ROC curve analysis

**Figure 4:**
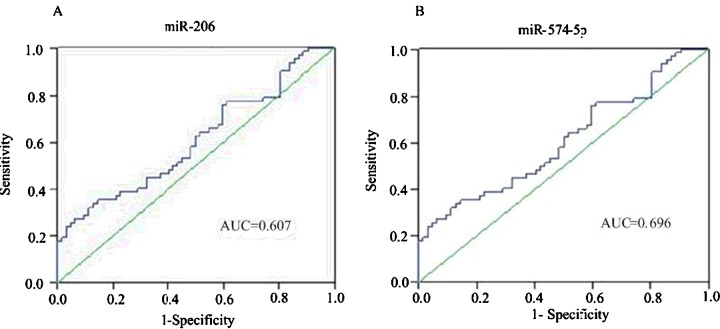
ROC curves for the ability of plasma levels of the selected two individual miRNAs The figure depicts calculated ROC curve and respective AUC values for *miR-206* and *miR-574-5p* (**A** and **B**), which exhibited good accuracy in differentiating CAD patients from matched healthy controls.

ROC curve and the area under the curve (AUC) can be used as a diagnostic method for evaluation of the accuracy of the indicators. To evaluate the predictive value of up-regulated miRNA for CAD, we calculated ROC curve for each of the two miRNAs with the AUC value. We obtained the following AUC values: *miR-206*, 0.607 (95% CI, 0.508–0.706) and *miR-574-5p*, 0.696 (95% CI, 0.609–0.787) respectively (Figures 4A and 4B). Our results demonstrated that these two miRNAs have a great potential to provide sensitive and specific diagnostic value.

## DISCUSSION

CAD is caused by atherosclerosis and other risk factors involved in coronary heart disease [24]. As CAD is among the most frequent causes of illness and death, an early diagnosis is essential. Several previous studies have indicated that there is a potential of plasma miRNAs as valuable biomarkers for CAD. For example, Fichtlscherer et al. [25] performed miRNAs arrays for serum or plasma of controls and CAD patients, and found that cardiac muscle-enriched miRNAs (*miR-133* and *miR-208a*) were overexpressed in patients as compared with controls. Additionally, a distinct miRNA profile of up-regulation for *miR-106b/25* cluster, *miR-17/92a* cluster, *miR-21/590-5p* family, *miR-126* and *miR-451* was observed in the plasma of patients with typical unstable angina and angiographically documented CAD, as compared with individuals with noncardiac chest pain (control group) [26]. Moreover, a recent study showed that *miR-1*, *miR-122*, *miR-126*, *miR-133a*, *miR-133b* and *miR-199a* were positively regulated in both stable and unstable angina patients, whereas *miR-337-5p* and *miR-145* exhibited an up-regulation only in stable or unstable angina patients respectively, compared with controls [27]. Condorelli, Wronska and Kudumula summarized the role of miRNA in cardiovascular disease separately. There is an emerging role of miRNAs in cardiac arrhythmias [15] and in CV disease in general [16,17]. These observations suggest that plasma miRNAs may be useful for not only the prediction but also the improvement of the diagnostic accuracy in CAD. Moreover, Santulli et al. [28] performed a miRNA-based approach to prevent restenosis through inhibiting proliferating vascular smooth muscle cells (VSMCs).

The gene for human *miR-206* (hsa-miR-206) is localized on chromosome 6 in a bicistronic cluster together with the gene for *miR-133b*, the later refers to a skeletal muscle-specific myomiR [29,30]. Various studies indicate that besides the skeletal muscle-specific character, *miR-206* may also play a pivotal role in cardiovascular diseases. The first study linking *miR-206* to the heart was conducted by Shan et al. [31]. The authors showed that in a mouse model of myocardial infarction, levels of *miR-1* and *miR-206* are increased in infarcted tissue. Limana et al. [32] demonstrated that an increase in *miR-206* was also observed in a rat model of heart failure and was even more prominent in mice treated with high mobility group box-1 protein (HMGB1).

In human, the *miR-574* gene is intronic. Initially, *miR-574-5p* was thought to be hosted by the first intron of the gene encoding *Noxp20* on human chromosome 4 [33]. Interestingly, alterations in the expression of *miR-574-5p* have been found to be associated with a variety of diseases, including cardiovascular diseases. Boštjančič et al. [34] found that both *miR-574-3p* and *miR-574-5p* showed up-regulation in infarcted heart tissue compared with corresponding remote myocardium in human myocardial infarction, as well as to healthy human hearts.

In the present study, we systematically determined the expression levels of plasma miRNAs in CAD patients, and our data firstly identified two plasma miRNAs (*miR-206* and *miR-574-5p*) which significantly up-regulated in CAD patients compared with control subjects. ROC analysis showed that the two miRNAs have a great potential to provide sensitive and specific diagnostic value. However the mechanism of how the two miRNAs are up-regulated is not clear yet.

## CONCLUSIONS

In summary, the present study provided insight into plasma levels of miRNAs in patients with CAD compared with healthy controls and demonstrated their potential as biomarkers for CAD. Validation of the changes in miRNA expression observed here in larger studies will be a necessary step to confirm their candidacy as biomarkers and therapeutic targets. We believe that further elucidation of the role of these miRNAs in the pathogenesis and progression of CAD will contribute to our understanding of the disease process and lead to new therapeutic and preventative strategies although they need more validation in future studies.

## Abbreviations

AUC: area under the curve
CAD: coronary artery disease
CV: cardiovascular
HDL-C: high-density lipoprotein cholesterol
LDL-C: low-density lipoprotein cholesterol
ROC: receiver operating characteristic
qRT-PCR: quantitative real-time PCR
